# Up-Regulation of microRNA-424 Causes an Imbalance in AKT Phosphorylation and Impairs Enteric Neural Crest Cell Migration in Hirschsprung Disease

**DOI:** 10.3390/ijms24076700

**Published:** 2023-04-04

**Authors:** Ze Xu, Yingnan Yan, Beilin Gu, Wei Cai, Yang Wang

**Affiliations:** 1 Department of Pediatric Surgery, Xinhua Hospital, School of Medicine, Shanghai Jiao Tong University, Shanghai 200092, China; 2 Shanghai Key Laboratory of Pediatric Gastroenterology and Nutrition, Shanghai 200092, China; 3 Shanghai Institute for Pediatric Research, Shanghai 200092, China

**Keywords:** miR-424, Hirschsprung disease, *RICTOR*, enteric neural crest cells, AKT phosphorylation

## Abstract

Insights into the role of microRNAs (miRNAs) in disease pathogenesis have made them attractive therapeutic targets, and numerous miRNAs have been functionally linked to Hirschsprung disease (HSCR), a life-threatening genetic disorder due to defective migration, proliferation, and colonization of enteric neural crest cells (ENCCs) in the gut. Recent studies have demonstrated that miR-424 strongly inhibits migration in a variety of cell types and its potential target *RICTOR* is essential for neural crest cell development. We therefore sought to interrogate how miR-424 and *RICTOR* contribute to the pathogenesis of HSCR. We utilized HSCR cases and human neural cells to evaluate the miR-424-mediated regulation of RICTOR and the downstream AKT phosphorylation. We further developed an ex vivo model to assess the effects of miR-424 on ENCC migration and proliferation. Then, single-cell atlases of gene expression in both human and mouse fetal intestines were used to determine the characteristics of *RICTOR* and *AKT* expression in the developing gut. Our findings demonstrate that miR-424 levels are markedly increased in the colonic tissues of patients with HSCR and that it regulates human neural cell migration by directly targeting *RICTOR*. Up-regulation of miR-424 leads to decreased AKT phosphorylation levels in a RICTOR-dependent manner, and this, in turn, impairs ENCC proliferation and migration in the developing gut. Interestingly, we further identified prominent *RICTOR* and *AKT* expressions in the enteric neurons and other types of enteric neural cells in human and mouse fetal intestines. Our present study reveals the role of the miR-424/RICTOR axis in HSCR pathogenesis and indicates that miR-424 is a promising candidate for the development of targeted therapies against HSCR.

## 1. Introduction

Hirschsprung disease (HSCR, MIM 142623), also known as congenital aganglionosis, is a life-threatening polygenic disorder, which is due to disturbances in the development of the enteric nervous system (ENS) [1]. This ENS developmental abnormality specifically arises from incomplete rostral–caudal migration, proliferation, or colonization of ENS progenitors—enteric neural crest cells (ENCCs)—in the gut [2]. HSCR is also the most frequent cause of functional intestinal obstruction with a population incidence of approximately 1/5000 live births (2.8/10,000 live births among Asian newborns), and it is classified according to the extent of aganglionosis into short-segment HSCR (S-HSCR, 80%, aganglionosis up to the upper sigmoid colon), long-segment HSCR (L-HSCR, 15%, aganglionosis up to the splenic flexure and beyond), and total colonic aganglionosis (TCA, 5%) forms [3,4].

HSCR shows high heritability (>80%), dramatic sex bias (approximately 4:1 affected males:females), high sibling recurrence risk (200-fold greater than the population), and non-Mendelian inheritance patterns in families [4], which are all hallmarks of a typical multifactorial genetic disorder. Hitherto, numerous genetic studies have identified dozens of HSCR-associated genes, where the major ones include *RET* (ret proto-oncogene) and *EDNRB* (endothelin receptor type B); yet, all these genes cumulatively explain only less than 10% of HSCR cases [3,5,6], suggesting that additional genes with low penetrance may also be involved in the etiology of HSCR. Growing evidence shows that *RICTOR* (RPTOR-independent companion of MTOR complex 2), an essential component of the mTORC2 complex, is required for embryonic development, particularly in neural crest cell development [7]. Indeed, Carson et al. have found a clear role for *Rictor* during neurodevelopment [8]. Interestingly, it has been recently demonstrated that Rictor can mediate cell migration and survival independent of mTORC2 [9,10], and knock-down of Rictor in endothelium impaired migration [11]. Agarwal et al. have shown that Rictor modulates cell migration by controlling RhoGDI2, a potent inhibitor of Rho proteins [12]. However, it is yet unclear how *RICTOR* contributes to the pathogenesis of HSCR.

MicroRNAs (miRNAs) are small regulatory molecules that function as negative regulators of gene expression at the post-transcriptional level by targeting the 3′ untranslated region of the mRNA and thereby modulate diverse biological processes, such as neuronal differentiation, development, plasticity, and survival [13]. miRNAs have also been observed to be dysregulated in a variety of pathological conditions, including cancer, neurodegenerative diseases, and cardiovascular diseases [14,15,16]. The ability of chosen miRNAs to target various mRNAs that are changed in pathological conditions makes these molecules interesting candidates as targets of therapeutics (in the form of anti-miRNAs) or as therapeutics (in the form of miRNA mimics) [17]. More recently, increasing evidence indicates that numerous miRNAs are altered in HSCR, and these regulators are involved in modulating the pathogenesis of HSCR by directly suppressing a variety of functional targets [18,19]. Of note, recent studies indicate that miR-424 plays important roles in regulating cell migration, and indeed overexpression of miR-424 inhibits the migration in various cell types, such as vascular smooth muscle cells, human umbilical vein endothelial cells, and glioblastoma cells [20,21,22].

In the present study, we show that miR-424 is markedly up-regulated in HSCR tissues, and this small molecule regulates the migration of human neural cells by directly targeting *RICTOR*. Overexpression of miR-424 leads to decreased levels of AKT phosphorylation and thereby inhibits the processes of cell migration. More importantly, our ex vivo model demonstrates that miR-424 plays a crucial role in regulating ENCC migration and proliferation in the developing gut. The characteristics of *RICTOR* and *AKT* expression are further determined by using single-cell atlases of gene expression in both human and mouse fetal intestines.

## 2. Results

### 2.1. RICTOR Correlates Inversely with miR-424 in HSCR

**Figure 1:**
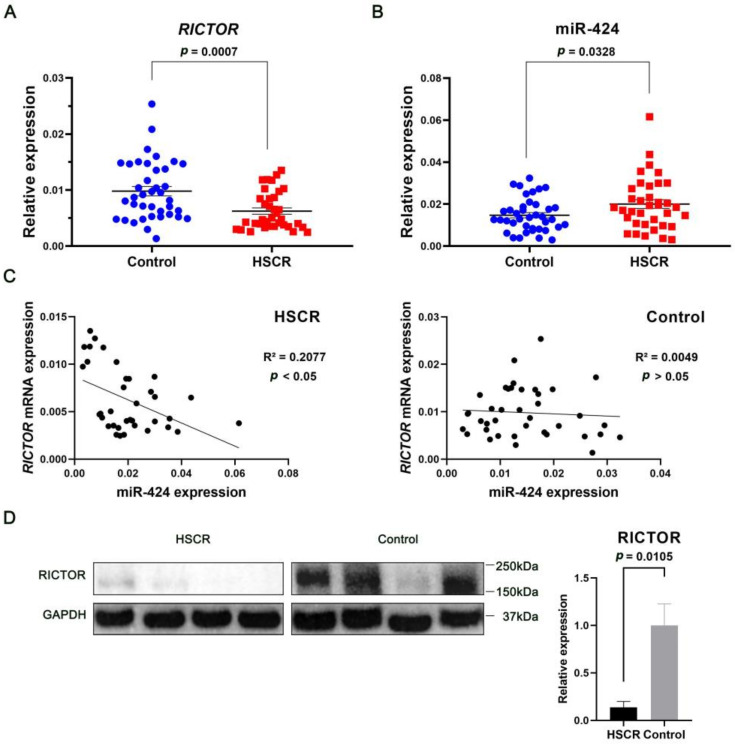
RICTOR, down-regulated in HSCR, inversely correlates with miR-424. (**A**,**B**) qRT-PCR analysis of *RICTOR* or miR-424 mRNA in the colonic tissues from patients with HSCR (*n* = 36) and normal controls (*n* = 39). The mRNA expressions of *RICTOR* and miR-424 were normalized to ACTB and U6, respectively. (**C**) Correlation between the levels of *RICTOR* and miR-424. The expression levels were quantified as shown in (**A**,**B**). (**D**) Western blot analysis of RICTOR protein in the colonic tissues from HSCR cases (*n* = 4) and controls (*n* = 4). Relative protein levels were normalized to GAPDH. Error bar = SEM (standard error of the mean).

To ascertain the role of *RICTOR* in the pathogenesis of HSCR, we first examined the expression of *RICTOR* in patients with HSCR and normal controls. In contrast to controls, we found that RICTOR was dramatically downregulated at both the mRNA and protein levels in HSCR tissues (Figure 1A,D). Moreover, we identified *RICTOR* as one of the highly ranked putative targets for miR-424 according to several target prediction algorithms such as TargetScan 7.1 (https://www.targetscan.org/vert_71/ (accessed on 18 February 2021)) and BCmicrO (http://compgenomics.utsa.edu/gene/gene_1.php (accessed on 18 February 2021)). To assess whether RICTOR reduction can be attributed to the elevated miR-424 level in HSCR, we evaluated the relative expression of miR-424 in HSCR cases and controls. As shown in Figure 1B, miR-424 expression was markedly higher in the HSCR group compared to the control group. We also noticed that the increased levels of miR-424 expression in HSCR tissues inversely correlated with the expression of *RICTOR* mRNA (Figure 1C left panel).

### 2.2. MiR-424 Directly Targets RICTOR in Human Neural Cells

**Figure 2:**
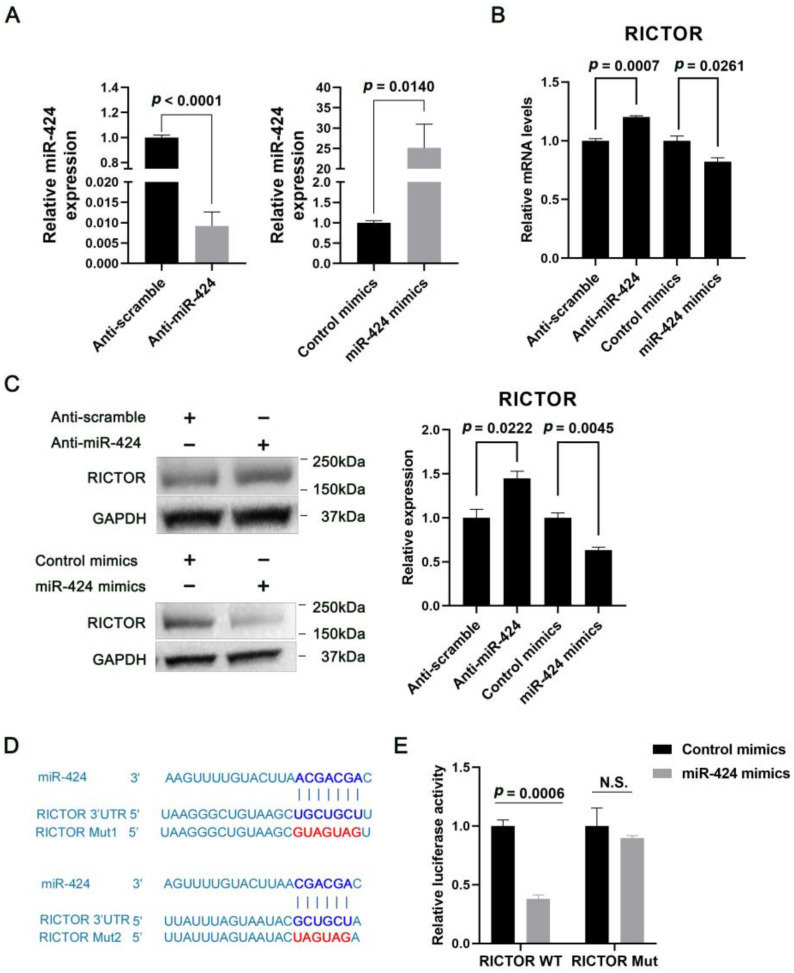
miR-424 directly targets RICTOR in human neural cells. (**A**–**C**) The effects of miR-424 on RICTOR mRNA and protein expression in human SH-SY5Y cells. qRT-PCR and Western blotting were used to assess the mRNA and protein expressions 72 h after transfection with control mimics, miR-424 mimics, anti-scramble, or anti-miR-424. Relative mRNA and protein levels were normalized to U6/ACTB (miR-424/RICTOR) and GAPDH, respectively. Representative blots are shown. (**D**) Predicted miR-424 binding sites within the 3′UTR of RICTOR. The mutated sequences in the miR-424 binding sites are highlighted in red. (**E**) Relative luciferase activities of the wild-type (RICTOR WT) or double mutant (RICTOR Mut) pGL3 promoter vectors co-transfected with either miR-424 mimics or control mimics into SH-SY5Y cells. Error bar = SEM (standard error of the mean); N.S., not significant; *n* = 3. WT, wild type; Mut, mutant.

To test whether human RICTOR is regulated by miR-424, we assessed its expression in human SH-SY5Y neuroblastoma cells transfected with synthetic miR-424 mimics or inhibitors. We achieved a transfection efficiency of approximately 90–95% for SH-SY5Y cells (Supplementary Figure S1). miR-424 overexpression strongly reduced the levels of RICTOR mRNA and protein, as detected by qRT-PCR and Western blotting, respectively, whereas inhibition of miR-424 significantly increased these levels (Figure 2A–C).

MiR-424 exhibits potential for the base-pairing with RICTOR mRNA, based on the extended 5′ 7-mer-seed and an additional 3′ binding site (Figure 2D). To validate the direct binding and regulation of RICTOR by miR-424, we further cloned a fragment of human RICTOR 3′ UTR containing the predicted miR-424 binding site to a pGL3 promoter vector, and co-transfected this construct with either miR-424 mimics or control mimics into SH-SY5Y cells. Compared with the control mimics, co-transfection of miR-424 mimics with wild-type RICTOR 3′ UTR significantly reduced the reporter gene activity, suggesting that miR-424 downregulates RICTOR expression (Figure 2E, RICTOR WT *p* = 0.0006). To identify the binding site of miR-424 in the RICTOR 3′ UTR, we carried out a similar luciferase activity assay with a mutated construct, and found this mutation dramatically abrogated the miR-424 regulation of the RICTOR 3′ UTR (Figure 2E, RICTOR Mut). These results clearly show that miR-424 binds directly to RICTOR 3′ UTR and represses RICTOR expression.

### 2.3. MiR-424 Modulates Human Neural Cell Migration in a RICTOR-Dependent Manner

**Figure 3:**
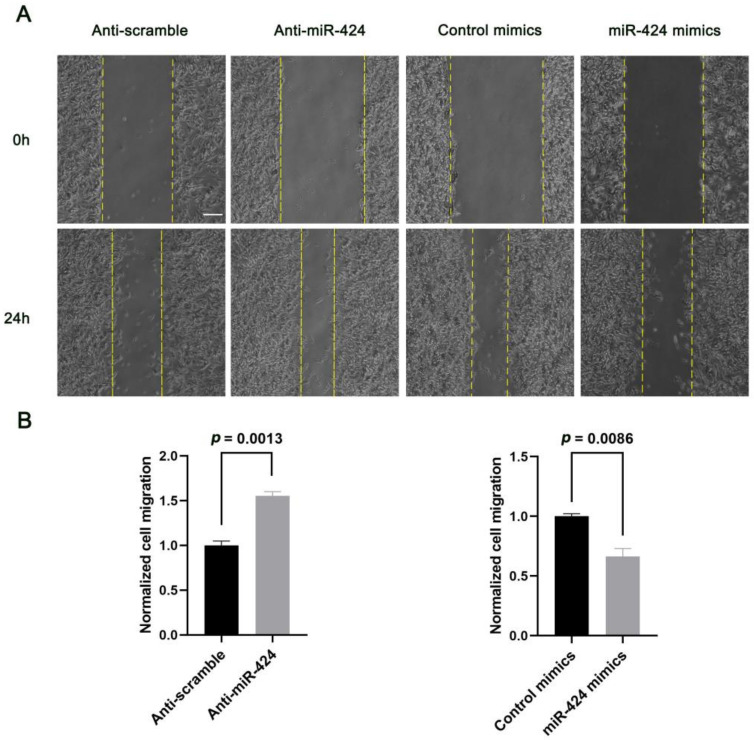
The effects of miR-424 on the migration of human neural cells. (**A**) Wound healing assay on SH-SY5Y cells: 72 h after transfection with control mimics, miR-424 mimics, anti-scramble, or anti-miR-424, the confluent transfected SH-SY5Y cells were scratch wounded. An additional 24 h was required for incubation, and images were taken at 0 h and 24 h. Scale bar = 200 μm. (**B**) Quantification of cell migration of SH-SY5Y cells. Cell migration was normalized to anti-scramble- or control mimic-treated cells. Error bar = SEM (standard error of the mean); *n* = 3.

**Figure 4:**
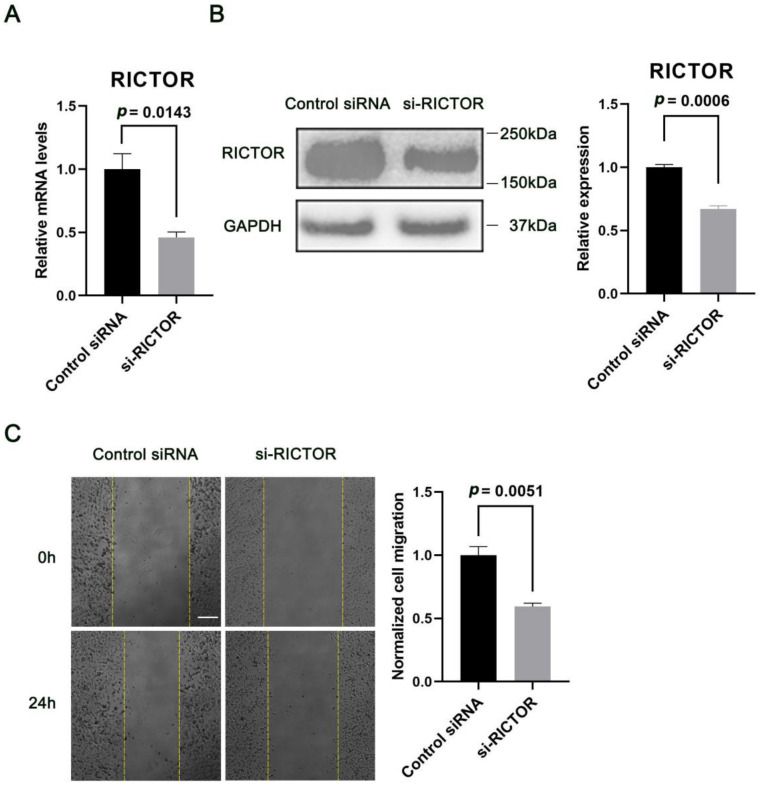
Down-regulation of RICTOR by siRNA and its effects on human neural cell migration. (**A**,**B**) Expression levels of RICTOR mRNA and protein in human SH-SY5Y cells. qRT-PCR and Western blotting were used to evaluate the mRNA and protein expressions 72 h after transfection with control siRNA or siRNA cognate to RICTOR. (**C**) Knock-down of RICTOR inhibits the migration of human neural cells. The wound healing assay was conducted as described in Figure 3. Scale bar = 200 μm. Error bar = SEM (standard error of the mean); *n* = 3.

**Figure 5:**
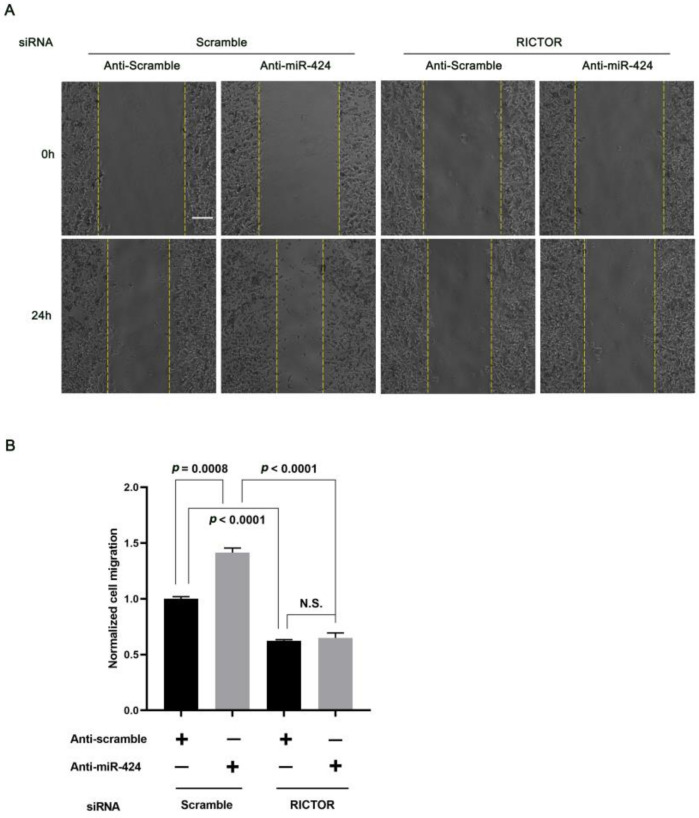
miR-424 regulates the migration of human neural cells in a RICTOR-dependent manner. (**A**) Knock-down of RICTOR rescues the anti-miR-424-induced enhancement in cell migration. SH-SY5Y cells were co-transfected with anti-scramble or anti-miR-424, and control siRNA or siRNA cognate to RICTOR for 72 h. The wound healing assay was carried out as described in Figure 3. Scale bar = 200 μm. (**B**) Quantification of human neural cell migration. Cell migration was normalized to anti-scramble-treated cells. Error bar = SEM (standard error of the mean); N.S., not significant; *n* = 3.

Next, we examined the effects of miR-424 on the migration of human neural cells by overexpressing or inhibiting miR-424 in human SH-SY5Y cells. Interestingly, over-expression of miR-424 in SH-SY5Y cells resulted in a significant decrease in cell migration (*p* = 0.0086) 24 h after confluent transfected SH-SY5Y cells were linearly scratch wounded while miR-424 inhibition markedly enhanced cell migration (*p* = 0.0013) (Figure 3). Additionally, we sought to understand how RICTOR affected the miR-424-mediated regulation of neural cell migration. Thus, we first transfected SH-SY5Y cells with either control siRNA or siRNA cognate to RICTOR for 72 h. Knock-down of RICTOR with the cognate siRNA significantly hindered the cell migration process (*p* = 0.0051) (Figure 4). SH-SY5Y cells were then co-transfected with miR-424 inhibitor and RICTOR-specific siRNA followed by wound healing assays. We found that the RICTOR-targeting siRNA rescued the effects of miR-424 on neural cell migration (Figure 5), suggesting that RICTOR is the key target that mediates miR-424 activity in the regulation of human neural cell migration.

### 2.4. MiR-424 Regulates AKT Phosphorylation through RICTOR-Dependent Mechanism

**Figure 6:**
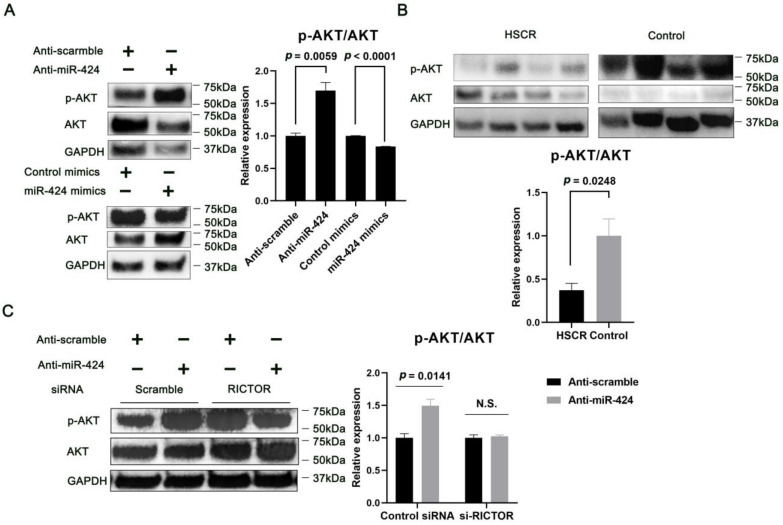
miR-424 modulates AKT phosphorylation via RICTOR. (**A**) The effects of miR-424 on AKT phosphorylation in human neural cells. SH-SY5Y cells were transfected with miR-424 mimics, anti-miR-424, or the corresponding controls. AKT phosphorylation at Ser473 (p-AKT) was assessed by Western blotting 72 h post-transfection. Representative blots are present. GAPDH serves as a loading control. Error bar = SEM (standard error of the mean); *n* = 3. (**B**) Western blot analysis of AKT phosphorylation in the colonic tissues from patients with HSCR (*n* = 4) and controls (*n* = 4). Error bar = SEM. (**C**) Knock-down of RICTOR rescues the anti-miR-424-induced increase in AKT phosphorylation. SH-SY5Y cells were co-transfected with anti-scramble or anti-miR-424, and control siRNA or siRNA cognate to RICTOR for 72 h. Representative blots show the levels of AKT phosphorylation. Error bar = SEM; N.S., not significant; *n* = 3.

miR-424 has been previously implicated in regulating the AKT-dependent signaling pathway [23,24], and of note, AKT phosphorylation promotes cell migration in a variety of cell types, including neural crest cells [25,26,27]. We therefore questioned whether miR-424 could have an impact on the phosphorylation of AKT in human neural cells. As shown in Figure 6A, the levels of AKT phosphorylated at S473, the main active form of AKT [28], was reduced by miR-424 mimics, and conversely, increased by the miR-424 inhibitor in human neural cells. Importantly, AKT phosphorylation at S473 was markedly decreased in HSCR tissues compared to normal control tissues (Figure 6B).

To further interrogate whether the effects of miR-424 on AKT phosphorylation were mediated by RICTOR, we co-transfected SH-SY5Y cells with miR-424 inhibitor and RICTOR-targeting siRNA, and observed that siRICTOR rescued the effects of miR-424 on AKT phosphorylation (Figure 6C). Our findings show that RICTOR mediates the regulation of AKT phosphorylation by miR-424 in human neural cells.

### 2.5. MiR-424 Regulates the Development of Enteric Neural Crest Cell Ex Vivo

**Figure 7:**
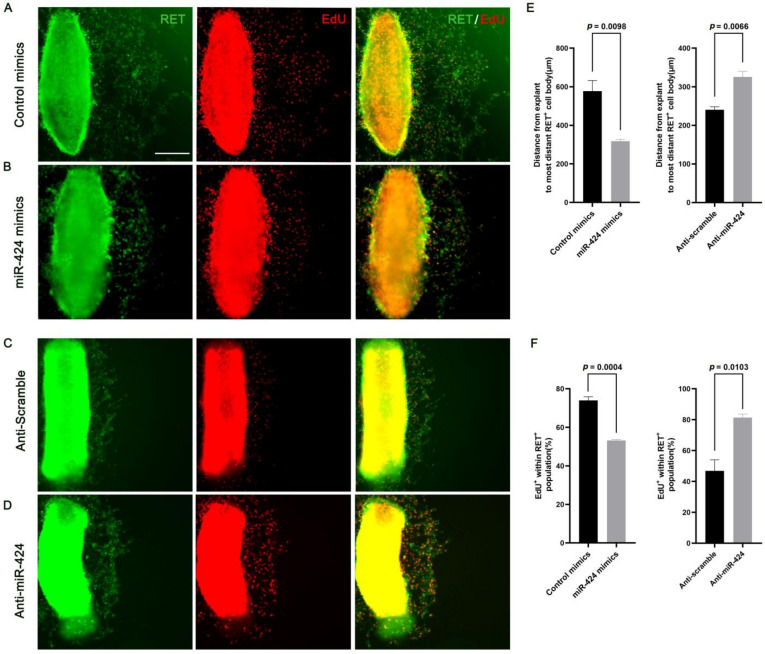
miR-424 regulates ENCC migration and proliferation in explant cultures. (**A**–**D**) Representative views of E12.5 midgut explants that were cultured for 24 h and immunostained for RET and EdU. The explants are at the left of each image. Scale bar = 200 μm. (**E**) Quantitative analysis of the distance traveled by the RET-expressing cells furthest from the edges of the explant after 24 h in culture. (**F**) Quantification of EdU labeling index within the RET-expressing population after 24 h in culture. Error bar = SEM (standard error of the mean); *n* = 3.

Since disturbances in ENCC migration along the developing gut can result in Hirschsprung disease, we next interrogated the role of miR-424 in modulating ENCC development. In order to assess ENCCs moving on a migration-permissive surface, we cultured E12.5 midgut slices on fibronectin in the presence of glial cell-derived neurotrophic factor (GDNF), a potent chemoattractant for ENCCs [29]. miR-424 overexpression significantly hindered RET+ cell migration out of the intestinal explants (Figure 7A,B,E), and reduced DNA synthesis, as shown by the number of EdU+ cells (Figure 7A,B,F). On the contrary, inhibition of miR-424 dramatically promoted migration of RET+ cells out of the explants (Figure 7C–E) and also markedly boosted ENCC proliferation (EdU+ cells) (Figure 7C,D,F). Collectively, our ex vivo findings allow us to conclude that in the developing gut, miR-424 plays a crucial role in regulating ENCC migration and proliferation, which is also essential for ENCCs to efficiently colonize the distal intestine [30].

### 2.6. Interrogation of the Cell Type Specificity of RICTOR and AKT Expression in ENS Development

**Figure 8:**
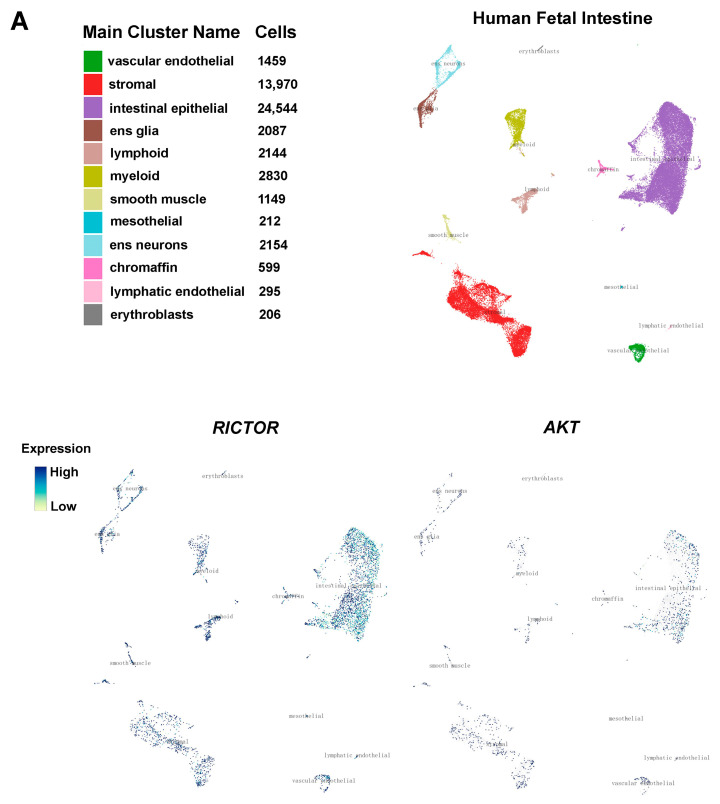

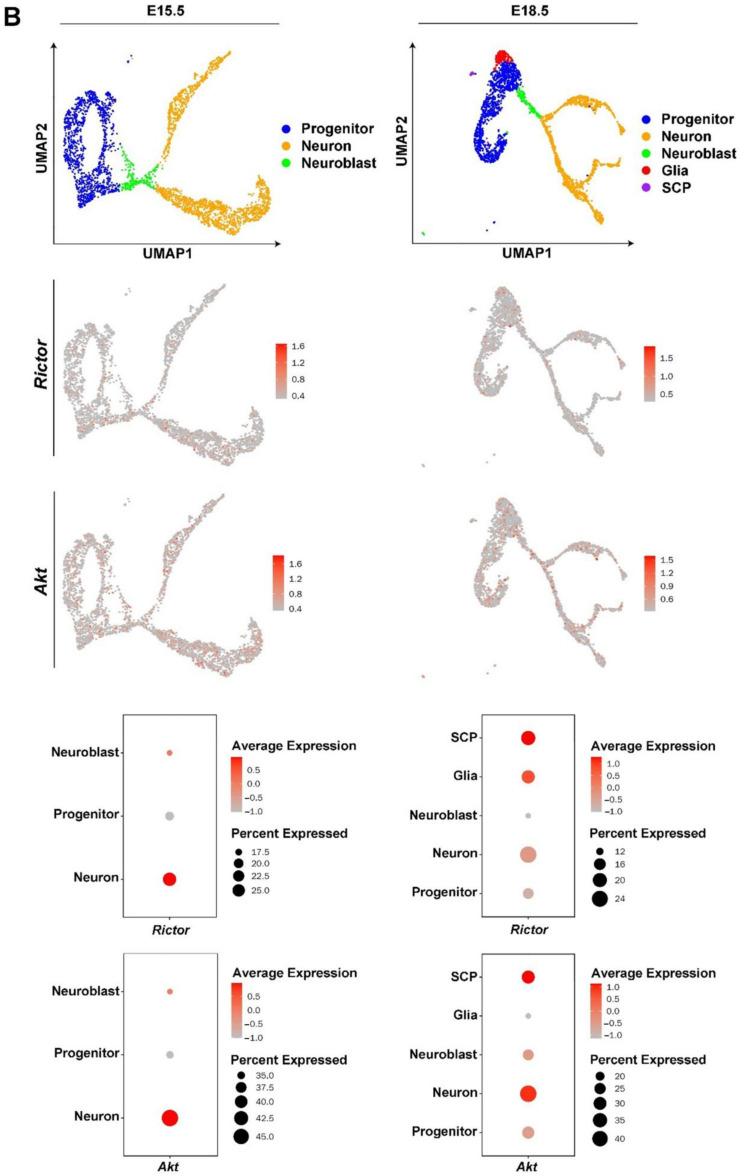
Interrogation of the cell type specificity of *RICTOR* and *AKT* in ENS development. (**A**) Uniform Manifold Approximation and Projection (UMAP) visualization and marker-based annotation of cells from the human fetal intestine colored by cell type (left panel). Plots in the right panel were colored by the normalized expression of cell-type-specific *RICTOR* and *AKT* in the human fetal intestine. (**B**) Expression of *Rictor* and *Akt* in different cell populations detected by scRNA-seq in mouse embryos at E15.5 and E18.5. UMAP indicates clusters corresponding to progenitors, neuroblasts, Schwann cells precursors (SCPs), enteric neurons, and glia. Feature plot and dot plot present the expression of *Rictor* and *Akt* and the percentage of cells in a cluster expressing *Rictor* or *Akt* and color bars show expression level with a maximum cut-off at the 90th percentile.

To determine the characteristics of *RICTOR* and *AKT* expression in the development of the enteric nervous system (ENS), we first utilized single-cell atlases of gene expression from human fetal intestines obtained during midgestation [31], which also helped us better understand the in vivo gene expression program underlying the specification of human cell types in a developing gut. Of note, in the human fetal intestine, we found that *RICTOR* was expressed at high levels in both ENS neurons and ENS glia, and *AKT* also showed a noticeable expression in these two types of cells, albeit at a lower level than *RICTOR* (Figure 8A). Meanwhile, we analyzed scRNA-seq data sets of the whole ENS isolated from the small intestine of Wnt1-Cre; R26R-Tomato mice at E15.5 and E18.5 [32]. Interestingly, both *Rictor* and *Akt* were expressed in a variety of ENS cell types at E15.5 and E18.5, including enteric progenitors, neurons, and glial populations (Figure 8B). Altogether, these scRNA-seq data sets indicated a potential role of *RICTOR* and *AKT* in ENS development.

## 3. Discussion

Numerous human diseases have been linked to aberrant expression of specific miRNAs, which frequently explains the severe pathogenic effects associated with disease initiation and/or progression. However, functions and therapeutic potentials of miRNAs dysregulated in Hirschsprung disease have not yet been fully elucidated. In this study, we unveiled that miR-424 was significantly up-regulated in patients with HSCR. Meanwhile, in a search for direct functional targets for miR-424, we recruited multiple target prediction algorithms and identified *RICTOR* as one of the highly ranked putative targets for miR-424. According to our findings, up-regulation of miR-424 accounts for the reduced expression of RICTOR that was observed in HSCR patients.

miR-424 has been reported for a broad range of human disorders, such as neurodegenerative diseases, pulmonary arterial hypertension, and various tumors [21,33,34]. More interestingly, miR-424 directly participates in the regulation of cell migration via multiple targets and signaling pathways. Wang et al. pointed out that LAMC1, the target of miR-424, could inhibit the angiogenesis and migration of HUVECs (human umbilical vein endothelial cells) by repressing the LAMC1-mediated Wnt/β-catenin signaling pathway [22]. On the other hand, a recent study showed that overexpression of miR-424 strongly suppressed cell proliferation and migration rate in glioblastoma cells, by targeting genes such as *KRAS*, *RAF1*, and *MAP2K1*, from the epidermal growth factor receptor (ERBB) signaling pathway [21]. In the present study, we, for the first time, demonstrated that miR-424 overexpression significantly slows down the migration of human neural cells. RICTOR can modulate cell migration [10] and several lines of our evidence show that miR-424 directly regulates its target RICTOR (Figure 2), and we subsequently found that the effects of miR-424 on human neural cell migration could be rescued by siRNA-mediated knock-down of RICTOR (Figure 3, Figure 4 and Figure 5), highlighting the important role of RICTOR in the miR-424-mediated regulation of cell migration.

More recent findings have shown that Akt phosphorylation facilitates the process of neural crest cell migration, and indeed it has been proven that miR-424 is involved in the AKT-dependent signaling pathway [24,27]. Interestingly, our study showed that up-regulation of miR-424 led to a markedly decreased level of AKT phosphorylation. In addition, we also demonstrated that the effects of miR-424 on AKT phosphorylation are mediated by RICTOR in human neural cells. On the other hand, the regulatory mechanism of AKT phosphorylation is intricate, and it has been recently demonstrated that knock-down of Rictor in vivo leads to mTORC2 inhibition [35,36], which can cause an increased level of p-Akt, and these findings were further confirmed in our present study (Figure 6C). The AKT family is comprised of three homologous kinases, AKT1, AKT2, and AKT3, and each of the AKT isoforms exhibits different tissue specificities, with AKT1 being abundantly expressed in both the human and rodent nervous systems, where Akt signaling is crucial for neurodevelopment and neuroplasticity [37,38]. Chadha et al. have recently identified a reduction in the phosphorylation of AKT (at serine 473) in the dorsolateral prefrontal cortex in patients with schizophrenia [39]. More importantly, a recent study has shown that AKT signaling can be regulated by a class IIb lysine deacetylase, histone deacetylase 6, in human neural progenitor cells [38]. On the other hand, D-cysteine can actually modulate the proliferation of neural progenitor cells by inhibiting AKT signaling [40]. Consistent with all these lines of evidence, our present findings suggest the possibility that miR-424-mediated regulation of AKT phosphorylation might be involved in the etiology of HSCR.

The explant, as an ex vivo model, has drawn particular attention since it conserves important in vivo characteristics, such as all types of cells and complex tissue architecture. More importantly, the explant technique allows us to subject fragments of organs derived from a single donor to various treatments and to control the environmental conditions to which the specific tissue is exposed, and thereby improving the reproducibility of the different possible processes shown in vivo by providing a better picture of the complex morphology found in the whole animal [41,42]. In this study, we established an intestinal explant which represents a relevant and sensitive model to investigate the effects of miR-424 on ENCC migration and proliferation since both of these processes are crucial to ENS development. Indeed, up-regulation of miR-424 strongly inhibits ENCC migration out of intestinal explants and also markedly reduces ENCC proliferation (Figure 7). Moreover, it is important to note that cultured intestinal explants appeared to have a good viability for up to 48 h in an environment of 95% O_2_ and 5% CO_2_ [43]. This ex vivo explant model not only helps us simplify the experimental setup when compared to an in vivo model, but also provides a dynamic and quantitative measurement of the miR-424-mediated regulation of ENCC migration and proliferation in the developing gut. On the other hand, additional in vivo models will definitely be needed in the future to advance our understanding of the miR-424-mediated regulatory mechanism in HSCR.

**Figure 9:**
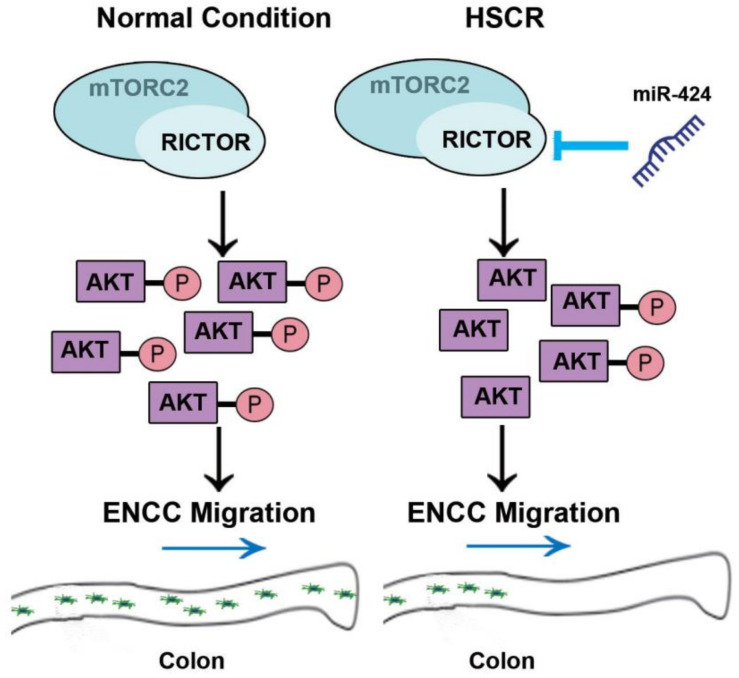
Schematic illustration of miR-424-mediated regulation of AKT phosphorylation and ENCC migration. Up-regulation of miR-424, observed in HSCR tissues, results in decreased RICTOR expression and thereby inhibits AKT phosphorylation. This, in turn, impairs ENCC proliferation and migration, consequently contributing to the pathogenesis of HSCR.

Of interest, we have for the first time identified decreased expression of RICTOR at both the mRNA and protein levels in the normoganglionic dilated segment of the HSCR colon, suggesting that the modulation of RICTOR may change during the progression of Hirschsprung disease. Indeed, dysregulated RICTOR has been linked to neuronal dysfunction and severe neurologic manifestations [8,44]. On the other hand, down-regulation of Rictor can actually impact mTORC2 downstream signaling targets, which thereby lead to long-term alterations in the central nervous system [45]. In the meantime, we observed a significant down-regulation of AKT phosphorylation in the colonic tissues of HSCR patients when compared to control tissues (Figure 6). According to the previous studies regarding the effects of AKT phosphorylation on promoting neural crest cell migration [27], our present results raise the possibility that the down-regulation of AKT phosphorylation may obstruct the migration process of enteric neural crest cells, leading to the induction of HSCR phenotypes. With all these findings, we then sought to interrogate the characteristics of RICTOR and AKT expression in the developing gut using single-cell atlases of gene expression. Since the ENS develops from highly motile streams of enteric stem cells and phenotypically distinct neurons are generated at different embryonic time windows, developing tissues may offer better opportunities to study the in vivo emergence and differentiation of different cell types [31,46,47]. Meanwhile, scRNA-seq has facilitated the mapping of organ development at an unprecedented resolution and has unveiled previously unidentified disease-related phenotypes in the gut [48,49,50]. Interestingly, our study showed prominent *Rictor* and *Akt* expressions in a variety of murine ENS cell types, particularly in enteric neurons. More importantly, we found significant *RICTOR* expression in the enteric neurons of the human fetal intestine by using the single-cell atlases which profile gene expression in 5 million cells [31]. *AKT* also exhibits a discernible expression in human fetal enteric neurons (Figure 8A); moreover, Akt signaling has recently been demonstrated to play a key role in ENS precursor development [51]. Figure 9 shows a schematic overview of the regulation of miR-424/RICTOR and the downstream events that result in reduced Akt phosphorylation and inhibition of ENCC proliferation and migration.

Taken together, our present study indicates a critical role of miR-424 and its direct target RICTOR in the risk of Hirschsprung disease. Understanding the molecular mechanisms modulating HSCR pathogenesis is of utmost importance for developing new therapies for HSCR, and we here show that additional complexity stems from post-transcriptional modulation and post-translational modification. Since the miR-122 inhibitor miravirsen has become the first “breakthrough” miRNA-targeted drug to enter human clinical trials [52], many more are in different stages of clinical development for a variety of complex human disorders. Given our findings, miR-424 is a promising candidate for therapeutic strategies for HSCR. Meanwhile, the growing body of evidence, coupled with identifying critical miRNAs involved in HSCR, will undoubtedly advance our understanding of this disease and make miRNA therapeutics a clinical reality.

## 4. Materials and Methods

### 4.1. Participants

In this study, we recruited 83 subjects, including 40 cases with HSCR (31 males and 9 females) and 43 controls (31 males and 12 females). The mean ages were 1.05 ± 1.93 years in the HSCR group and 1.34 ± 1.41 years in the control group. All the participants recruited in this study were biologically unrelated individuals of Han Chinese ancestry. Supplementary Table S1 shows the detailed characteristics of the study population. The patients with HSCR were diagnosed based on histological examination of a biopsy or surgical resection material for the absence of ganglion cells. We used the normoganglionic dilated segment of the colonic tissue from patients with HSCR, and randomly enrolled controls from the participants who had no history of chronic constipation. This study was performed in accordance with the principles of the Declaration of Helsinki. The study was reviewed and approved by the ethics committee of Xinhua Hospital of Shanghai Jiao Tong University School of Medicine. Participants or their parents provided written informed consent after the procedure had been fully explained.

### 4.2. Cell Cultures and Transfections

The human neuroblastoma SH-SY5Y cells, which have been widely used as a model for HSCR, were maintained in Dulbecco’s Modified Eagle Medium (DMEM)/Nutrient Mixture F-12 supplemented with 10% fetal bovine serum (FBS), and 1% penicillin and streptomycin, and were passaged by trypsinization. Cell cultures were maintained at 37 °C and 5% CO_2_. The miR-424 inhibitor, scramble control, miR-424 mimics, control mimics, siRNA for RICTOR, and control siRNA were purchased from GenePharma (Shanghai, China). By using qRT-PCR to evaluate the three candidate siRNAs for RICTOR silencing, we found that si-RICTOR-2 and si-RICTOR-3 were more effective compared to si-RICTOR-1 (Supplementary Figure S2) and thus si-RICTOR-2 was used in the current study. Lipofectamine 2000 (Thermo Fisher Scientific, Waltham, MA, USA) was used to perform transfections of siRNAs, and miRNA inhibitors and mimics (at 50 nM final concentration) (Supplementary Table S2). SH-SY5Y cells were transfected according to the manufacturer’s instructions.

### 4.3. RNA Isolation and Quantitative Real-Time PCR (qRT-PCR)

Total RNA was extracted from cell cultures and tissues with Invitrogen TRIzol (Thermo Fisher Scientific, USA) following the manufacturer’s instructions. For miRNA, we used miRNA First Strand cDNA Synthesis (Tailing Reaction) (Sangon Biotech, Shanghai, China), and the miRNA levels were normalized to the uniformly expressed U6 snRNA. For mRNA, we employed RevertAid First Strand cDNA Synthesis Kit and PowerUp SYBR Green Master Mix (Thermo Fisher Scientific, USA), and assessed the mRNA expression by qRT-PCR on a QuantStudio System (Thermo Fisher Scientific, USA). All primers used are listed in Supplementary Table S3. Threshold cycles (Cts) were generated automatically, and the relative expressions were present as 2^−ΔCt^. The mRNA levels were normalized to ACTB.

### 4.4. Western Blot Analysis

Protein lysates were harvested and the concentrations were determined by PierceTM BCA Protein Assay Kit (Thermo Fisher Scientific, USA). Western blot analysis was carried out using a standard procedure, as previously described [14]. The following primary antibodies were used following the manufacturer’s instructions: RICTOR (Bethyl Laboratories, #A300-459A, TX, USA, 1:2000), AKT (Cell Signaling Technology, #9272S, MA, USA, 1:2000), p-AKT (S473) (Cell Signaling Technology, #4060S, MA, USA, 1:2000), and GAPDH (Santa Cruz, #sc-25778, TX, USA, 1:2000). Antibody detection was carried out using HRP-linked anti-rabbit IgG antibody (Cell Signaling Technology, #7074S MA, USA), followed by the ECL reaction (Millipore, Burlington, MA, USA). The signals from a fluorescent Western blot were quantified using the ImageJ software.

### 4.5. Validation of miR-424 Targets by Luciferase Reporter Assay

A fragment of RICTOR 3′ UTR or a double-mutated sequence of the predicted target sites was inserted into the Xbal site of the pGL3 promoter vector to validate the predicted miR-424 binding site (Genechem, Shanghai, China). The constructs were co-transfected with either miR-424 mimics or control mimics (at a final concentration of 50 nM) into the SH-SY5Y cells using Lipofectamine 2000 (Thermo Fisher Scientific, USA). We employed the Dual-Luciferase^®^ Reporter Assay System (Promega, Madison, WI, USA) to quantify luciferase luminescence (Renilla and firefly) 48 h after transfections. 

### 4.6. Wound Healing Assay

Linear scratches were created in the monolayer of SH-SY5Y cells using a P200 pipette tip. The incubation was continued for another 24 h. The ImageJ software was used to examine the images which were taken at 0 and 24 h. The following equation was used to measure cell migration: (0 h wound area − 24 h wound area)/0 h wound area × 100. The cell migration test was independently performed three times, and the values for cell migration under each condition were normalized to the relevant control.

### 4.7. Mouse Strain and Primary ENCC Culture

C57BL/6N mice (Charles River Laboratories) were used for developing the ex vivo model. Specifically, male mice were housed with female mice in the same cage at a ratio of 1:2. The presence of a vaginal plug was a sign of successful mating, which was considered to be 0.5 days of pregnancy and recorded as E0.5. All mouse experimental procedures and protocols were reviewed and authorized by the Animal Care and Use Committees of Xinhua Hospital of Shanghai Jiao Tong University School of Medicine. Animal care was strictly carried out in accordance with institutional guidelines. For cultures of embryonic intestinal explants, approximately 500 μm slices of E12.5 midgut were cultured in chamber slides (Thermo Fisher Scientific, USA), which were precoated with 250 μg/mL fibronectin for 30 min. Explant cultures were maintained in serum-free DMEM/F12 medium supplemented with penicillin/streptomycin and 50 ng/mL GDNF for 24 h (37 °C, 5% CO_2_). At 4 h after plating, cultures were transfected with miR-424 mimics, control mimics, anti-miR-424, and anti-scramble using Lipofectamine 2000 (Thermo Fisher Scientific, USA). When appropriate, we added 10 μM EdU (Beyotime, Shanghai, China) 5 h before fixation. 

### 4.8. Immunohistochemistry

E12.5 midgut slices were washed with PBS, fixed with 4% paraformaldehyde (PFA) for 30 min at 25 °C, and permeabilized/blocked for 1 h at 25 °C in TBST (Tris-buffered saline with 0.1% Triton-X 100) and 5% goat serum. The slices were then incubated with primary antibodies (rabbit anti-RET, Cell Signaling Technology, #3223S, MA, USA, 1:200) overnight at 4 °C, and further incubated with fluorophore-conjugated secondary antibodies (Alexa Fluor^®^ 488 goat anti-rabbit, Yeasen, #33106ES60, Shanghai, China, 1:400) for 1 h at 25 °C. For the cell proliferation assay, the cells were immunostained with BeyoClick™ EdU Cell Proliferation Kit (Beyotime, Shanghai, China) following the manufacturer’s instructions. All images were taken with a Leica DMI6000B microscope. Cell migration (RET+ cells) and proliferation (EdU+ cells) were assessed using the ImageJ software.

### 4.9. Single-Cell RNA Sequencing Data Sets and Analysis

For human fetal intestines, we used single-cell atlases of gene expression from a previous study [31], and examined the data using the DESCARTES platform, which is based on a three-level single-cell combinatorial indexing assay for gene expression (sci-RNA-seq3) to 121 human fetal samples [31]. For mice, the single-cell RNA sequencing (scRNA-seq) data sets, deposited under accession GSE149524 at Gene Expression Omnibus, were analyzed as previously described [32]; these data sets included the whole ENS isolated from the small intestine of Wnt1-Cre; R26R-Tomato mice at E15.5 and E18.5 [32].

### 4.10. Statistical Analysis

GraphPad Prism V.6 (GraphPad Software, CA, USA) was used to perform the comparison among multiple groups using an unpaired, 2-tailed Student’s *t*-test. All images were quantified with the ImageJ Software (Version 1.49j, NIH). All experiments have been replicated at least 3 times. *p* < 0.05 was considered to be statistically significant, and the data are shown as mean ± SEM (standard error of the mean).

## Data Availability

Data supporting the findings of this work are available in the article/Supplementary Materials.

## Supplementary Materials

The following supporting information can be downloaded at: https://www.mdpi.com/article/10.3390/ijms24076700/s1.
