# Supplementary material for: Up-Regulation of microRNA-424 Causes an Imbalance in AKT Phosphorylation and Impairs Enteric Neural Crest Cell Migration in Hirschsprung Disease
Source: Int J Mol Sci. 2023 Apr 4;24(7):6700. doi: 10.3390/ijms24076700 (PMC10094892; doi:10.3390/ijms24076700)
Supplement: Supplementary file 1 [file ijms-24-06700-s001.zip › ijms-2221282-supplementary.pdf]

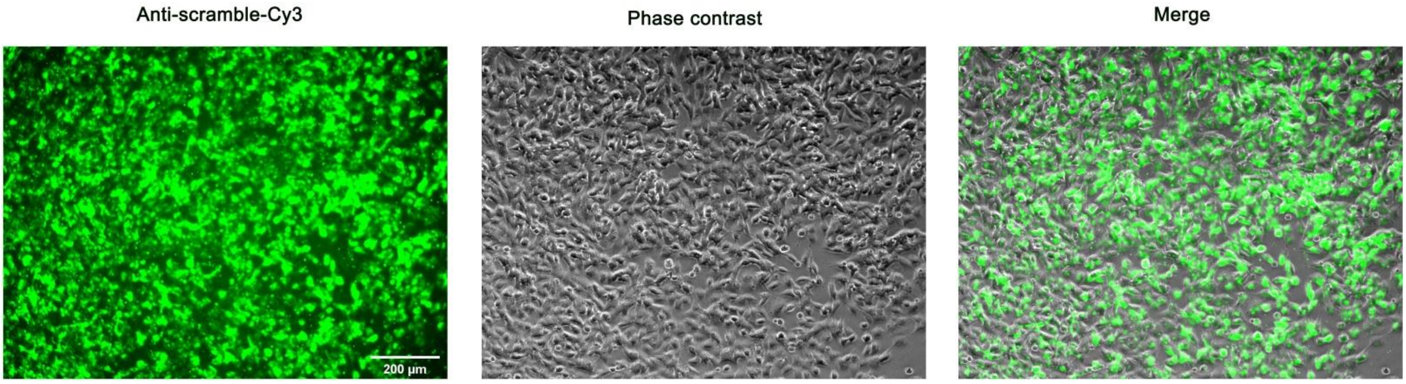

Figure S1. The transfection efficiency of SH-SY5Y cells transfected with Cy3-labeled anti-scramble. Majority of SH-SY5Y cells (approximately 90-95%) were positive for Cy3-labeled anti-scramble (anti-scramble-Cy3) after transfection.

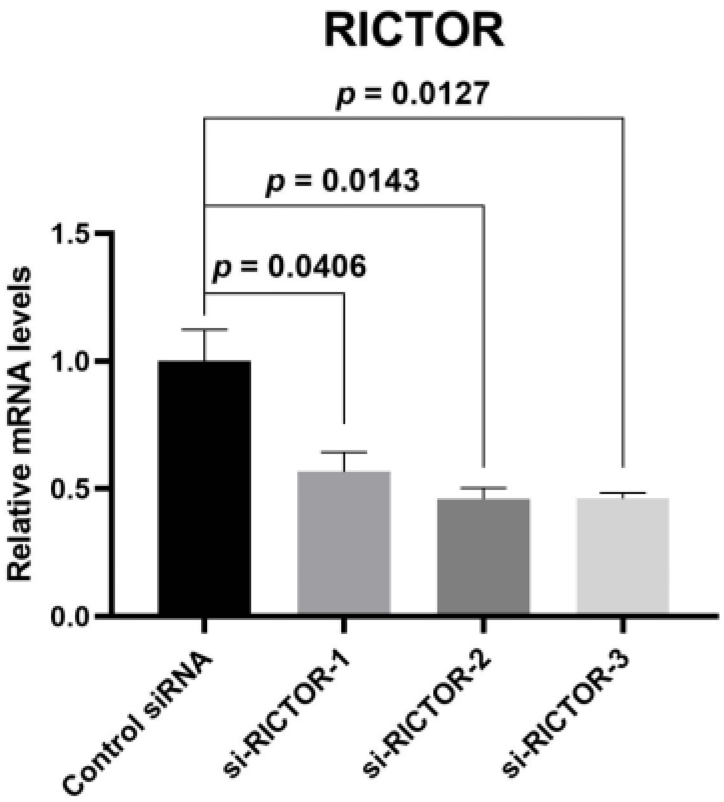

Figure S2. The effectiveness regarding knock-down of RICTOR with candidate siRNAs in human SH-SY5Y cells. The mRNA expression levels were quantified by using qRT-PCR 72 hours after transfection with control siRNA, or the three candidate siRNAs cognate to RICTOR (i.e. si-RICTOR-1, si-RICTOR-2 and si-RICTOR-3). Relative mRNA levels were normalized to ACTB. Error bar = SEM (standard error of the mean); N.S., not significant; n=3.

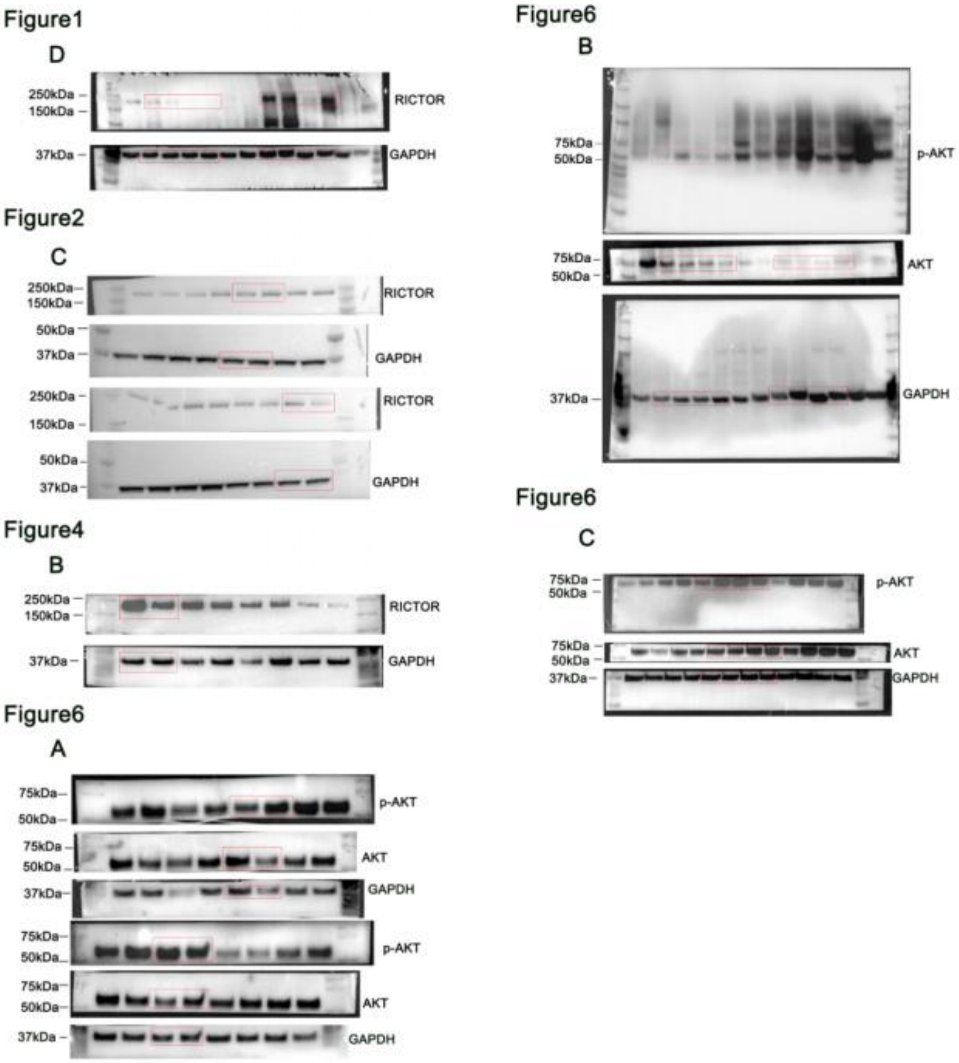

Figure S3. Raw images of the immunoblotting experiments.

Table S1. Demographic and clinical characteristics of the subjects

Characteristics	Case (n=40)	Control (n=43)
Male	31	31
Female	9	12
Age ± SD	1.05 ± 1.93	1.34 ± 1.41
S-HSCR, No. (%)	32 (80.0)	
L-HSCR, No. (%)	6 (15.0)	
TCA, No. (%)	2 (5.0)	

HSCR = Hirschsprung disease,

L-HSCR = long-segment HSCR, S-HSCR = short-segment HSCR,

TCA = total colonic aganglionosis

Table S2. Synthetic oligonucleotides used for transfections.

Name		Sequence 5' to 3'
miR-424 mimics	Sense	CAGCAGCAAUUCAUGUUUUGAA
	Anti-sense	CAAAACAUGAAUUGCUGCUGUU
miR-424 inhibitor	Sense	UUCAAAACAUGAAUUGCUGCUG
RICTOR siRNA-1	Sense	GGUGGGUUUAUGAGUAUAUTT
	Anti-sense	AUAUACUCAUAAACCCACCTT
RICTOR siRNA-2	Sense	GCGGUUAGCUUUAUUAAAUTT
	Anti-sense	AUUUAAUAAAGCUAACCGCTT
RICTOR siRNA-3	Sense	CCUGCCUAUACACCUUUAUTT
	Anti-sense	AUAAAGGUGUAUAGGCAGGTT

Table S3. Primers used for qRT-PCR reactions.

Gene	Accession No.		Primer sequence 5' to 3'
RICTOR	NM_152756.5	F	TGGATCTGACCCGAGAACCT
		R	TCCTCATAGTGAAAGCCCAGT
ACTB	NM_001101.5	F	CACCTTCTACAATGAGCTGCGTGTG
		R	ATAGCACAGCCTGGATAGCAACGTAC
MIR424	NR_029946.1	F	CAGCAGCAATTCATGTTTTGAA
		R	Provided by Sangon Biotech (Shanghai, China)
RNU6-1	NR_004394.1	F	Provided by Sangon Biotech (Shanghai, China)
		R	
